# Plasma microRNA Expression and Micronuclei Frequency in Workers Exposed to Polycyclic Aromatic Hydrocarbons

**DOI:** 10.1289/ehp.1307080

**Published:** 2014-03-14

**Authors:** Qifei Deng, Suli Huang, Xiao Zhang, Wangzhen Zhang, Jing Feng, Tian Wang, Die Hu, Lei Guan, Jun Li, Xiayun Dai, Huaxin Deng, Xiaomin Zhang, Tangchun Wu

**Affiliations:** 1 State Key Laboratory of Environmental Health (Incubating), School of Public Health, Tongji Medical College, Huazhong University of Science and Technology, Wuhan, Hubei, China; 2 Institute of Industrial Health, Wuhan Iron and Steel Corporation, Wuhan, Hubei, China; * These authors contributed equally to this work.

## Abstract

Background: Ubiquitous polycyclic aromatic hydrocarbons (PAHs) have been shown to alter gene expression patterns and elevate micronuclei (MN) frequency, but the underlying mechanisms are largely unknown. MicroRNAs (miRNAs) are key gene regulators that may be influenced by PAH exposures and mediate their effects on MN frequency.

Objectives: We sought to identify PAH-associated miRNAs and evaluate their associations with MN frequency.

Methods: We performed a two-stage study in healthy male coke oven workers to identify miRNAs associated with PAH exposures quantified using urinary monohydroxy-PAHs and plasma benzo[*a*]pyrene-*r*-7,*t*-8,*c*-10-tetrahydrotetrol-albumin (BPDE–Alb) adducts. In the discovery stage, we used Solexa sequencing to test differences in miRNA expression profiles between pooled plasma samples from 20 exposed workers and 20 controls. We then validated associations with eight selected miRNAs in 365 workers. We further evaluated associations between the PAH-associated miRNAs and MN frequency.

Results: In the discovery stage, miRNA expression profiles differed between the exposed and control groups, with 68 miRNAs significantly down-regulated [fold change (FC) ≤ –5] and 3 miRNAs mildly up-regulated (+2 ≤ FC < +5) in the exposed group. In the validation analysis, urinary 4-hydroxyphenanthrene and/or plasma BPDE–Alb adducts were associated with lower miR-24-3p, miR-27a-3p, miR-142-5p, and miR-28-5p expression (*p* < 0.030). Urinary 1-hydroxynaphthalene, 2-hydroxynaphthalene, 2-hydroxyphenanthrene, and the sum of monohydroxy-PAHs were associated with higher miR-150-5p expression (*p* < 0.030). These miRNAs were associated with higher MN frequency (*p* < 0.005), with stronger associations in drinkers (*p*_interaction_ < 0.015).

Conclusions: Associations of PAH exposures with miRNA expression, and of miRNA expression with MN frequency, suggest potential mechanisms of adverse effects of PAHs that are worthy of further investigation.

Citation: Deng Q, Huang S, Zhang X, Zhang W, Feng J, Wang T, Hu D, Guan L, Li J, Dai X, Deng H, Zhang X, Wu T. 2014. Plasma microRNA expression and micronuclei frequency in workers exposed to polycyclic aromatic hydrocarbons. Environ Health Perspect 122:719–725; http://dx.doi.org/10.1289/ehp.1307080

## Introduction

Polycyclic aromatic hydrocarbons (PAHs) are ubiquitous environmental pollutants that are generated primarily through incomplete combustion of carbon-containing materials. One of the major sources of environmental PAHs is industrial activities that constantly emit high concentrations of PAHs, such as tar distillation and coke production. Substantial epidemiological evidence suggests that long-term exposure to PAH-rich emissions is associated with a higher lung cancer risk in exposed workers (International Agency for Research on Cancer 2005). Before exerting their carcinogenic effects, PAHs are metabolically activated to form stable PAH–DNA adducts and cause DNA oxidation, eventually leading to DNA damage (Xue and Warshawsky 2005). DNA damage can result in chromosome aberrations and genetic instability, and might eventually trigger cancers. Micronucleus (MN) frequency is extensively used as a biomarker of chromosomal damage, genome instability, and cancer risk (El-Zein et al. 2006; Fenech 2007). Previous studies have reported that occupational exposure to PAHs is associated with higher MN frequency (Liu et al. 2006; Pavanello et al. 2008). However, the underlying mechanisms still need to be clarified.

In experimental systems, PAHs have been shown to alter the expression patterns of genes that are relevant to the carcinogenic effects of PAHs (Hewitt et al. 2007; Staal et al. 2006). Effects on microRNA (miRNA) expression have been proposed as a mechanism through which environmental exposures might affect gene expression (Jirtle and Skinner 2007; Schembri et al. 2009). miRNAs are a class of small noncoding RNAs that function as gene regulators by base pairing with the 3´-untranslated regions of target mRNAs and leading to translational repression or degradation of target mRNAs (Ambros 2004). There is growing evidence that exposure to environmental pollutants may significantly disrupt miRNA expression patterns (Bollati et al. 2010; De Flora et al. 2012; Izzotti et al. 2009; Jardim et al. 2009; Schembri et al. 2009). However, few epidemiological studies have examined potential effects of PAH exposures on miRNA expression profiles. miRNAs play crucial roles in a broad range of physiological and pathological processes, including the processes that may affect genetic damage levels, such as DNA damage response (Lal et al. 2009; Pothof et al. 2009) and oxidative stress (Sangokoya et al. 2010). Previously, miRNAs were shown to regulate cellular responses to ultraviolet-induced genetic damage *in vitro* (Pothof et al. 2009). However, no studies have demonstrated effects of miRNAs on PAH-related chromosome damage specifically.

Circulating miRNAs in plasma have the potential to serve as stable noninvasive biomarkers of physiological and pathological conditions (Mitchell et al. 2008). Thus in the present study, we sought to identify plasma miRNAs that are associated with PAH exposures by genome-wide miRNA sequencing and subsequent validation in healthy male coke oven workers with well-characterized PAH exposures. We further evaluated associations between the PAH-associated miRNAs and MN frequency.

## Materials and Methods

*Airborne PAH monitoring*. We collected airborne samples from different workplaces in the coke plant of a steel mill located in southern China and determined the concentrations of 16 PAHs using high-performance liquid chromatography (Li et al. 2012).

*Study subjects*. In a previous study (Li et al. 2012), we enrolled 1,333 coke oven workers (1,126 males and 207 females) who were employed at the coke plant for at least 1 year and worked at the top, side, and bottom of the coke ovens; adjunct workplaces (such as the blower operation room and recycling workshops); or in offices. The present study population is a subset of the previous study and consists of male workers 20–60 years of age. We excluded *a*) workers with a self-reported history of chronic diseases, including cancers, cardiopulmonary diseases, chronic inflammation, and hypertension; *b*) workers who reported taking any medicines in the preceding 3 months; and *c*) workers who did not provide urine and/or blood samples. Thus, a total of 391 workers participated in all parts of this study. After participants provided informed consent, we administered a questionnaire to collect information on demographic characteristics, smoking and drinking habits, medical history, and occupational experiences. Participants were considered smokers unless they had smoked an average of < 1 cigarette/day for < 1 year in their lifetime (nonsmokers), and considered drinkers unless they had drunk alcoholic beverages less than once each week for < 1 year in their lifetime (nondrinkers). Each worker donated approximately 20 mL morning urine and 5 mL fasting venous blood (~ 4 mL heparin-anticoagulated and ~ 1 mL EDTA-anticoagulated). This study was approved by the Medical Ethics Committee of the School of Public Health, Tongji Medical College.

*Study design*. Based on ambient concentrations of total PAHs measured in our previous study (Li et al. 2012), we classified 256 participants who worked in offices (mean ± SD, 1.13 ± 0.37 μg/m^3^) or at adjunct workplaces (3.72 ± 2.09 μg/m^3^) as the control group, and 135 workers working at the top of the coke oven (90.30 ± 69.51 μg/m^3^) or at the side and bottom of the coke oven (11.08 ± 7.29 μg/m^3^) as the exposed group. Because each of the urinary monohydroxy-PAHs (OH-PAHs) and plasma benzo[*a*]pyrene-*r*-7,*t*-8,*c*-10-tetrahydrotetrol-albumin (BPDE–Alb) adducts were significantly correlated with the sum of OH-PAHs (ΣOH-PAHs) (*p* < 0.001) and because the sum of correlation coefficients was the highest for ΣOH-PAHs (see Supplemental Material, Table S1), we used ΣOH-PAHs as the representative PAH internal exposure biomarker for sample selection for miRNA sequencing in the discovery stage. We selected 20 workers with higher ΣOH-PAHs from the exposed group, and 20 matched workers with lower ΣOH-PAHs from the control group. We intentionally frequency-matched the distribution of important general characteristics, including age (± 5 years), smoking status, pack-years of smoking (± 5 pack-years), drinking status, working years (± 2 years), and body mass index (BMI) (± 2), between these two groups to minimize their confounding effects on miRNA expression profiles. We prepared a 5-mL pooled plasma sample for each group that included 250 μL of plasma from each subject. We then subjected two plasma pools to miRNA sequencing and compared miRNA expression profiles between these two groups. To focus on the most likely related miRNAs in the validation stage, we selected miRNAs based on the following criteria: *a*) demonstrated at least a 5-fold lower or higher expression in the exposed group compared with the control group; *b*) expressed at least 50 copies in at least one group; and *c*) were found to be associated with PAH response, DNA damage, or other DNA damage-related mechanisms, such as oxidative stress, based on an extensive literature review. The remaining plasma of 14 subjects in the discovery stage (5 in the control group and 9 in the exposed group) was enough for quantitative reverse-transcription polymerase chain reaction (qRT-PCR) validation (≥ 200 μL); thus, these 14 subjects were also included in the validation stage.

For the miRNAs that were significantly associated with at least one PAH exposure biomarker, we further evaluated their associations with MN frequency.

*Urinary creatinine and OH-PAH measurements*. We measured urinary creatinine concentration, according to Jaffe’s colorimetric method, on an automated clinical chemistry analyzer. We measured 12 urinary OH-PAHs, 10 noncarcinogenic metabolites (1-hydroxynaphthalene, 2-hydroxynaphthalene, 2-hydroxyfluorene, 9-hydroxyfluorene, 1-hydroxyphenanthrene, 2-hydroxyphenanthrene, 3-hydroxyphenanthrene, 4-hydroxyphenanthrene, 9-hydroxyphenanthrene, and 1-hydroxypyrene) and 2 carcinogenic metabolites (6-hydroxychrysene and 3-hydroxybenzo[*a*]pyrene), by gas chromatography-mass spectrometry (Li et al. 2012). Limits of quantification (LOQ) were in the 0.1–1.4 μg/L range; for concentrations below the LOQ, we used 50% of the LOQ (see Supplemental Material, Table S2). The molar concentrations of OH-PAHs were calibrated by urinary creatinine and expressed as micromoles per millimole creatinine.

*Determination of plasma BPDE–Alb adducts*. We measured BPDE–Alb adducts in heparin-anticoagulated plasma using a sandwich enzyme-linked immunosorbent assay (Chung et al. 2010). We assayed each sample in duplicate. The average concentration of BPDE–Alb adducts for each sample was calibrated by plasma albumin and expressed as nanograms per milligram albumin. The LOQ was 1 ng/mg albumin; concentrations below the LOQ were replaced with 50% of the LOQ (see Supplemental Material, Table S2).

*Cytokinesis-block micronucleus (CBMN) assay*. Using fresh heparin-anticoagulated whole blood, with two duplicate slides for each subject, we performed a CBMN assay according to the standardized protocol developed by Fenech (2007). We microscopically examined 1,000 binucleated cells on each slide and identified the number of binucleated cells containing MN according to scoring criteria. The MN frequency of each subject was recorded as the mean number of CBMN cells per 1,000 binucleated cells.

*RNA isolation*. We isolated total RNA from the two EDTA-anticoagulated plasma pools collected in the discovery stage for Solexa sequencing; we also isolated total RNA from 200 μL EDTA-anticoagulated plasma from each subject in the validation stage, which was analyzed by qRT-PCR (mirVana PARIS miRNA Isolation Kit; Ambion, Austin, TX). For RNA isolation in the validation stage, we added *Canorhabditis elegans* miRNA cel-miR-39 (synthesized by QIAGEN, Germantown, MD, USA) to the denatured plasma samples to normalize the sample-to-sample variation in the isolation step.

*Solexa sequencing*. Purified small RNA molecules of < 30 bases were ligated with Solexa adaptors and reverse transcribed into cDNA. We used the purified cDNA for cluster generation and sequencing analysis using the Solexa Sequencer (Illumina, San Diego, CA). We then converted the generated image files into digital-quality data. After removing the adaptors, low-quality reads, and contaminated reads, we compared the clean reads with miRBase 18.0 to identify miRNAs. We calculated the normalized copy number for each miRNA by the equation (*C* × 10^6^)/*N*, where *C* is the number of reads mapped to individual miRNA, and *N* is the total number of aligned reads.

*qRT-PCR assay*. RNAs were reverse transcribed (TaqMan miRNA Reverse Transcription Kit) and then subjected to real-time PCR in duplicate using the TaqMan miRNA Assay Kit and an ABI Prism 7900HT Sequence Detection System (all from Applied Biosystems, Foster City, CA). The miRNA expression levels were normalized against cel-miR-39 and calculated by the 2^–ΔCt^ method, where Ct is the cycle threshold: Δ*C_t_* = *C_t_*_miRNA_ – *C_t_*_cel-miR-39_.

*Target gene prediction and enriched biological function analysis*. We used miRanda (http://www.microrna.org/microrna/home.do) and TargetScan (http://www.targetscan.org/) to predict the potential target genes of miRNAs, and we conducted Gene Ontology (GO) function enrichment analysis of these target genes using DAVID (Database for Annotation, Visualization and Integrated Discovery) Bioinformatics Resources, 6.7 (http://david.abcc.ncifcrf.gov).

*Statistical analyses*. Because 6-hydroxychrysene and 3-hydroxybenzo[*a*]pyrene were always below their LOQs, we excluded them from further analyses. The concentrations of the 10 remaining urinary OH-PAHs and plasma BPDE–Alb adducts were natural logarithm (ln) transformed, and the miRNA expression levels measured by qRT-PCR were log_2_ transformed. Thus, we used the transformed urinary OH-PAHs, plasma BPDE–Alb adducts, and miRNA expression levels in the statistical analyses. We evaluated the differences of general characteristics between different groups (control vs. exposed groups in the discovery stage, and discovery vs. validation) by Student’s *t*-test (for continuous variables) and chi-square test (for categorical variables). We analyzed between-group differences in urinary OH-PAHs and plasma BPDE–Alb adducts by multivariate covariance analysis, and MN frequency by Poisson regression analysis, with adjustment for several primary confounding factors, including age (continuous), smoking status (yes/no), pack-years of smoking (continuous), drinking status (yes/no), all working years (continuous), BMI (continuous), and/or workplace (control/exposed group), when appropriate.

In the discovery stage, we performed Hierarchical clustering for the miRNAs that showed differential expression in two groups using the PermutMatrix clustering tool (Caraux and Pinloche 2005). In the validation stage, we used multivariate linear regression models to evaluate associations between miRNA expression levels (as the dependent variable) and creatinine-corrected urinary OH-PAHs or plasma BPDE–Alb adducts, with adjustment for the above confounding variables. To compare the magnitude of these associations, we reported standardized coefficients (β_std_) that represent the estimated difference in log_2_-transformed miRNA expression (in SD units) associated with a 1-SD increase in the ln-transformed PAH exposure levels.

In previous work (Zhang X, Guan L, Huang K, Zhang WZ, Hu D, He YF, Li J, Lin DF, Guo YJ, Wu TC, unpublished data) and in the present study (see Supplemental Material, Table S3), OH-PAHs and BPDE–Alb adducts were significantly associated with MN frequency. Thus, to minimize the confounding effects of PAH exposure, we also adjusted for ln-transformed ΣOH-PAHs (continuous) and BPDE–Alb adducts (continuous) in Poisson regression models to evaluate the associations of log_2_-transformed miRNA expression levels (in SD units) with unstandardized MN frequency (as the dependent variable). The frequency ratio (FR) was calculated based on the equation *e*^βstd^ and represented the proportional changes of MN frequency due to 1-SD increase in log_2_-transformed miRNA expression. We evaluated the differences in the associations between miRNA and MN frequency in workers with different drinking status, smoking status, or age group by modeling interaction terms of (miRNA * stratum variables) in Poisson regression models. We carried out all data analyses using SPSS (version 12.0). Two-sided *p* < 0.05 was considered statistically significant.

## Results

*Subject characteristics*. As shown in Table 1, in the discovery stage, the distribution of age, smoking status, pack-years of smoking, drinking status, number of working years, and BMI were matched between the control group and exposed group (all *p* > 0.05), whereas the PAH exposure levels and MN frequencies were all significantly different between these two groups (all *p* ≤ 0.005). The distribution of general characteristics, most of the PAH internal exposure biomarkers (except for 1-hydroxynaphthalene, 3-hydroxyphenanthrene, and BPDE–Alb adducts), and MN frequency for the 40 subjects in the discovery stage were not significantly different from the other 351 workers in the validation stage (all *p* > 0.05).

**Table 1:** General characteristics, PAH exposure concentration, and MN frequency in the discovery and validation populations.

Variable	Discovery stage		*p-*Value	Validation population (*n* = 365)^*a*^	*p-*Value^*b*^
	Controls (*n* = 20)	Exposed (*n* = 20)			
General characteristic
Age (years)	39.85 ± 5.97	41.74 ± 7.82	0.394^*c*^	42.15 ± 8.12	0.314^*c*^
Smoking status [yes/no (% yes)]	15/5 (75.0)	14/6 (70.0)	0.723^*c*^	252/113 (69.0)	0.619^*c*^
Pack-years of smoking	10.69 ± 11.18	15.07 ± 15.13	0.305^*c*^	12.88 ± 15.14	0.965^*c*^
Drinking status [yes/no (% yes)]	6/14 (30.0)	9/11 (45.0)	0.327^*c*^	160/205 (43.8)	0.441^*c*^
No. of working years	18.55 ± 6.20	22.04 ± 8.59	0.149^*c*^	20.94 ± 9.59	0.720^*c*^
BMI	24.18 ± 3.30	23.85 ± 3.43	0.754^*c*^	24.10 ± 3.19	0.832^*c*^
PAH internal exposure biomarker^*d*^
1-Hydroxynaphthalene (× 10^–2^)	0.54 (0.44, 0.65)	4.28 (3.53, 7.32)	< 0.001^*e*^	1.11 (0.67, 1.82)	0.045^*e*^
2-Hydroxynaphthalene (× 10^–2^)	0.64 (0.33, 0.86)	3.68 (2.54, 6.15)	< 0.001^*e*^	1.13 (0.62, 1.68)	0.148^*e*^
2-Hydroxyfluorene (× 10^–3^)	2.19 (1.79, 3.65)	13.22 (8.22, 27.19)	< 0.001^*e*^	4.73 (2.96, 7.41)	0.450^*e*^
9-Hydroxyfluorene (× 10^–3^)	0.69 (0.06, 1.78)	7.15 (3.39, 19.84)	< 0.001^*e*^	2.72 (1.02, 5.87)	0.730^*e*^
1-Hydroxyphenanthrene (× 10^–3^)	0.79 (0.50, 1.51)	16.81 (9.93, 25.59)	< 0.001^*e*^	3.96 (1.93, 7.47)	0.557^*e*^
2-Hydroxyphenanthrene (× 10^–3^)	0.71 (0.52, 1.03)	6.30 (3.78, 12.11)	< 0.001^*e*^	1.46 (0.85, 2.77)	0.127^*e*^
3-Hydroxyphenanthrene (× 10^–3^)	1.19 (0.68, 1.62)	7.46 (3.45, 16.03)	< 0.001^*e*^	1.81 (1.01, 3.21)	0.031^*e*^
4-Hydroxyphenanthrene (× 10^–3^)	1.69 (0.32, 2.46)	4.00 (2.84, 12.98)	0.005^*e*^	1.73 (0.68, 3.31)	0.408^*e*^
9-Hydroxyphenanthrene (× 10^–3^)	1.21 (0.73, 1.78)	12.18 (10.22, 18.93)	< 0.001^*e*^	3.51 (2.00, 6.66)	0.963^*e*^
1-Hydroxypyrene (× 10^–2^)	0.68 (0.52, 0.90)	7.50 (4.95, 11.15)	< 0.001^*e*^	1.31 (0.82, 2.39)	0.052^*e*^
ΣOH-PAHs (× 10^–2^)	3.16 (2.59, 3.41)	21.09 (17.37, 37.44)	< 0.001^*e*^	6.12 (4.27, 9.43)	0.134^*e*^
BPDE–Alb	3.94 (3.43, 4.51)	6.33 (4.41, 10.01)	0.002^*e*^	4.20 (3.45, 5.17)	0.005^*e*^
Chromosome damage
MN frequency (per 1,000 cells)	2 (1,3)	4 (3,6)	< 0.001^*e*^	3 (2, 5)	0.092^*e*^
Values shown are mean ± SD, median (25th percentile, 75th percentile), or *n* (%). ^***a***^For all variables, ^*n*^ = 365. ^***b***^Comparisons between 40 workers in the discovery stage and the other 351 workers in the validation stage. ^***c***^*p*-Value determined by Student’s *t*-test for continuous variables and by chi-square test for categorical variables. ^***d***^Units for OH-PAHs are μmol/mmol creatine, and units for BPDE–Alb adducts are ng/mg albumin. ^***e***^Multivariate covariance analysis was performed for PAH internal exposure biomarkers, and Poisson regression analysis was performed for MN frequency, with adjustment for age, smoking status, pack-years of smoking, drinking status, number of working years, BMI, and/or workplace when appropriate.					

**Figure 1:**
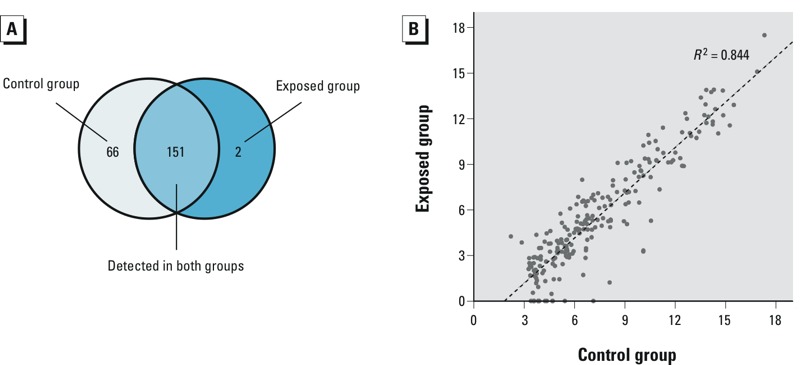
miRNA expression profiles in the discovery stage. (*A*) Distribution of miRNAs in the control group and the exposed group. (*B*) Scatter plots showing the relationship of the log_2_-transformed expression levels of miRNAs between the two groups.

**Figure 2:**
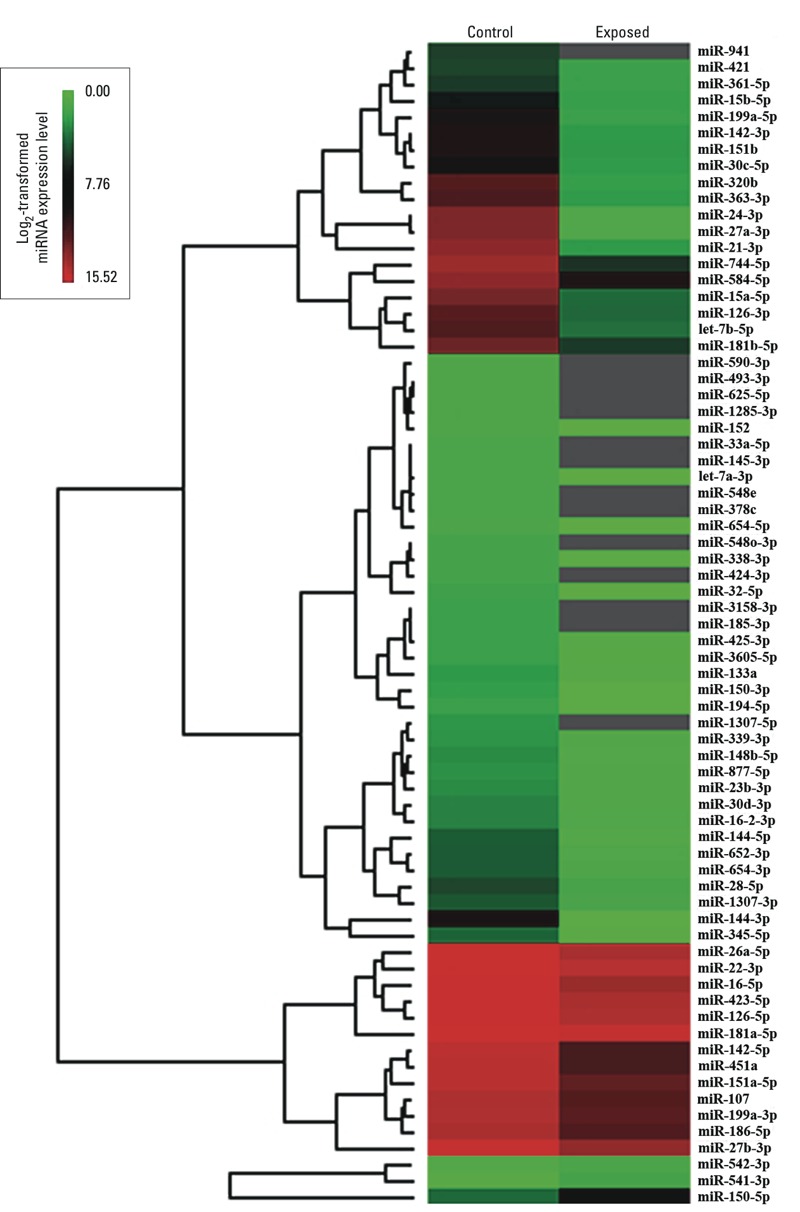
Heat map of the log_2_-transformed expression levels of 68 miRNAs significantly down-regulated (FC ≤ –5) in the exposed group compared with the controls, and 3 miRNAs (miR-542-3p, miR-541-3p, and miR-150-5p) mildly up‑regulated (+2 ≤ FC < +5) in the exposed group compared with the controls. Gray indicates no detection.

*miRNA expression profiles and miRNA selection for validation*. In the discovery stage, we detected a total of 217 and 153 miRNAs in the plasma pools of the control group and the exposed group, respectively (Figure 1A). The miRNA expression levels in these two groups were strongly correlated (*R*^2^ = 0.844) (Figure 1B). Notably, compared with the expression levels in the control group, 68 miRNAs demonstrated at least a 5-fold lower expression in the exposed group [all fold changes (FC) ≤ –5] (Figure 2), whereas no miRNA showed at least a 5-fold higher expression in the exposed group. Based on the miRNA selection criteria (see “Materials and Methods”), we selected 7 highly differentially expressed miRNAs from these 68 down-regulated miRNAs. Moreover, there were 3 miRNAs (miR-542-3p, miR-541-3p, and miR-150-5p) that showed at least a 2-fold higher expression in the exposed group than in the control group (+2 ≤ all FC < +5) (Figure 2). We also selected 1 miRNA (miR-150-5p) from these 3 mildly up-regulated miRNAs that expressed ≥ 50 copies in at least one group. The expression levels and the related functions of 8 selected miRNAs are shown in Table 2.

**Table 2:** Expression levels and related functions of the eight selected miRNAs.

miRNA	Discovery stage^*a*^		FC^*b*^	Validation stage^*c*^	Related functions	References
	Control	Exposed				
miR-24-3p	1,109	10	–110.9	0.221 (0.024, 1.193)	DNA damage response; ARNT regulation	Lal et al. 2009; Oda et al. 2012; Wang and Taniguchi 2013
miR-27a-3p	1,106	10	–110.6	0.247 (0.054, 0.924)	ROS-mediated repression; TFIIH regulation	Pathi et al. 2011; Portal 2011
miR-142-5p	5,901	470	–12.56	0.012 (0.003, 0.048)	Benzo[*a*]pyrene response	Halappanavar et al. 2011
miR-16-5p	24,442	2080	–11.75	0.622 (0.240, 1.659)	Regulation of DNA damage signaling in response to PAHs	Niziolek-Kierecka et al. 2012
miR-451a	5,493	475	–11.56	0.165 (0.077, 0.477)	Regulation of susceptibility to oxidative damage	Yu et al. 2010
miR-28-5p	131	18	–7.28	0.006 (0.001, 0.025)	Associated with frequent chromosomal alteration; antioxidant response	Wilting et al. 2013; Yang et al. 2011
let-7b-5p	532	83	–6.41	0.102 (0.028, 0.307)	Down-regulated by cellular stress; protecting cells from oxidant injury	Hou et al. 2012; Saleh et al. 2011
miR-150-5p	88	252	+2.86	0.015 (0.008, 0.037)	Benzo[*a*]pyrene response	Halappanavar et al. 2011
Abbreviations: ARNT, aryl hydrocarbon receptor nuclear translocator; ROS, reactive oxygen species; TFIIH, transcription factor II H. ^***a***^Mean copy numbers based on Solexa sequencing. ^***b***^Negative values indicate that miRNA expression was lower in the exposed group; positive values indicate that miRNA expression was higher in the exposed group. ^***c***^Values are median (25th percentile, 75th percentile) RT-PCR expression relative to cel-miR-39.						

*Identification of PAH-associated miRNAs.* In the validation stage, we used multivariable linear regression analysis to estimate confounder-adjusted associations of urinary OH-PAH concentrations and plasma BPDE–Alb adducts with the expression of eight selected miRNAs (Table 3). Urinary 4-hydroxyphenanthrene concentration was associated with significantly lower expression of miR-24-3p, miR-27a-3p, and miR-142-5p (all *p* < 0.030), with the strongest association estimated with miR-27a-3p [β_std_ = –0.141; 95% confidence interval (CI): –0.246, –0.037; *p* = 0.008]. Plasma BPDE–Alb adducts were associated with lower expression of miR-24-3p and miR-28-5p (all *p* ≤ 0.004), with the strongest association estimated with miR-28-5p (β_std_ = –0.180; 95% CI: –0.290, –0.071; *p* = 0.001). Notably, miR-24-3p was significantly associated with both 4-hydroxyphenanthrene (β_std_ = –0.117; 95% CI: –0.222, –0.013; *p* = 0.028) and BPDE–Alb adducts (β_std_ = –0.166; 95% CI: –0.277, –0.055; *p* = 0.004). miR-150-5p was the only selected miRNA that showed a mildly higher expression in the exposed group (+2 < FC < +5) in the discovery stage. In the validation stage, urinary 1-hydroxynaphthalene, 2-hydroxynaphthalene, 2-hydroxyphenanthrene, and ΣOH-PAHs were all associated with higher miR-150-5p expression (all *p* < 0.030), with the strongest association estimated with 1-hydroxynaphthalene (β_std_ = 0.190; 95% CI: 0.076, 0.303; *p* = 0.001). These five PAH-associated miRNAs were not significantly associated with drinking status, smoking status, or age (all *p* > 0.05) (see Supplemental Material, Table S4).

**Table 3:** Association of PAH exposure with miRNA expression level (as the dependent variable) [β_std_ (95% CI)].

PAH biomarker^*a*^	miR‑24‑3p^*b*^	*p*^*c*^	miR‑27a‑3p^*b*^	*p*^*c*^	miR‑142‑5p^*b*^	*p*^*c*^	miR‑16‑5p^*b*^	*p*^*c*^	miR‑451a^*b*^	*p*^*c*^	miR‑28‑5p^*b*^	*p*^*c*^	let‑7b‑5p^*b*^	*p*^*c*^	miR‑150‑5p^*b*^	*p*^*c*^
1-Hydroxy­naphthalene	–0.007 (–0.125, 0.110)	0.902	–0.024 (–0.138, 0.091)	0.686	0.039 (–0.075, 0.153)	0.503	0.059 (–0.056, 0.174)	0.317	0.074 (–0.040, 0.187)	0.202	0.033 (–0.082, 0.148)	0.576	0.089 (–0.026, 0.204)	0.130	0.190 (0.076, 0.303)	0.001
2-Hydroxy­naphthalene	–0.007 (–0.133, 0.118)	0.908	–0.041 (–0.164, 0.083)	0.517	0.018 (–0.105, 0.141)	0.776	0.078 (–0.047, 0.203)	0.219	0.089 (–0.034, 0.213)	0.156	0.013 (–0.111, 0.137)	0.839	0.080 (–0.046, 0.206)	0.214	0.153 (0.029, 0.277)	0.016
2-Hydro­xyfluorene	–0.058 (–0.163, 0.046)	0.274	–0.037 (–0.142, 0.067)	0.483	–0.074 (–0.178, 0.030)	0.161	–0.031 (–0.136, 0.075)	0.565	–0.033 (–0.137, 0.070)	0.527	0.043 (–0.062, 0.148)	0.424	0.043 (–0.063, 0.149)	0.423	0.013 (–0.093, 0.118)	0.813
9-Hydro­xyfluorene	–0.010 (–0.116, 0.096)	0.857	–0.040 (–0.145, 0.066)	0.461	–0.038 (–0.143, 0.067)	0.482	0.031 (–0.076, 0.138)	0.566	–0.008 (–0.113, 0.097)	0.874	0.037 (–0.069, 0.143)	0.491	0.041 (–0.067, 0.148)	0.459	0.050 (–0.057, 0.156)	0.361
1-Hydroxy­phenanthrene	0.037 (–0.075, 0.149)	0.517	–0.046 (–0.157, 0.065)	0.414	0.011 (–0.099, 0.121)	0.846	0.024 (–0.088, 0.136)	0.674	0.022 (–0.088, 0.132)	0.695	0.016 (–0.095, 0.128)	0.771	0.027 (–0.085, 0.139)	0.639	0.074 (–0.038, 0.185)	0.195
2-Hydroxy­phenanthrene	0.000 (–0.106, 0.107)	0.996	–0.004 (–0.111, 0.103)	0.945	–0.014 (–0.120, 0.092)	0.799	0.031 (–0.077, 0.139)	0.573	0.007 (–0.099, 0.114)	0.892	0.075 (–0.032, 0.182)	0.171	0.038 (–0.070, 0.146)	0.488	0.121 (0.014, 0.228)	0.026
3-Hydroxy­phenanthrene	–0.034 (–0.139, 0.072)	0.529	0.027 (–0.079, 0.134)	0.615	–0.007 (–0.112, 0.099)	0.901	–0.014 (–0.121, 0.093)	0.797	0.016 (–0.089, 0.121)	0.763	0.038 (–0.069, 0.144)	0.488	0.069 (–0.038, 0.176)	0.206	0.046 (–0.061, 0.152)	0.402
4-Hydroxy­phenanthrene	–0.117 (–0.222, –0.013)	0.028	–0.141 (–0.246, –0.037)	0.008	–0.121 (–0.225, –0.018)	0.022	–0.065 (–0.171, 0.040)	0.224	–0.035 (–0.139, 0.070)	0.516	–0.063 (–0.168, 0.043)	0.244	–0.029 (–0.134, 0.076)	0.588	–0.053 (–0.158, 0.053)	0.329
9-Hydroxy­phenanthrene	–0.063 (–0.168, 0.043)	0.244	–0.030 (–0.136, 0.076)	0.574	–0.019 (–0.125, 0.086)	0.719	0.006 (–0.101, 0.113)	0.917	0.004 (–0.101, 0.108)	0.946	0.004 (–0.103, 0.110)	0.944	0.000 (–0.107, 0.107)	0.998	0.086 (–0.021, 0.193)	0.114
1-Hydroxypyrene	–0.055 (–0.166, 0.056)	0.329	–0.087 (–0.196, 0.023)	0.121	–0.061 (–0.171, 0.048)	0.269	–0.009 (–0.120, 0.102)	0.878	0.017 (–0.092, 0.126)	0.759	–0.017 (–0.128, 0.093)	0.756	–0.027 (–0.138, 0.085)	0.638	0.091 (–0.020, 0.202)	0.107
ΣOH-PAHs	–0.030 (–0.143, 0.082)	0.593	–0.061 (–0.173, 0.050)	0.279	–0.017 (–0.128, 0.094)	0.758	0.035 (–0.078, 0.147)	0.544	0.015 (–0.096, 0.126)	0.793	0.014 (–0.098, 0.126)	0.810	0.048 (–0.066, 0.162)	0.407	0.150 (0.038, 0.261)	0.009
BPDE–Alb	–0.166 (–0.277, –0.055)	0.004	–0.084 (–0.194, 0.026)	0.134	–0.091 (–0.202, 0.019)	0.104	–0.050 (–0.163, 0.064)	0.389	–0.029 (–0.139, 0.081)	0.607	–0.180 (–0.290, –0.071)	0.001	–0.030 (–0.140, 0.080)	0.590	–0.067 (–0.182, 0.047)	0.249
^***a***^Values were ln-transformed. ^***b***^Values were log_2_-transformed. ^***c***^Multivariable linear regression analysis with adjustment for age, smoking status, pack-years of smoking, drinking status, working years, workplace, and BMI.																

*PAH-associated miRNAs and MN frequency*. We then investigated whether there were associations between the expression of the five PAH-associated miRNAs and MN frequency. As shown in Table 4, the five PAH-associated miRNAs were all associated with higher MN frequency (all *p* < 0.005), with the strongest association estimated for miR-24-3p (FR = 1.152; 95% CI: 1.086, 1.222; *p* = 2.59 × 10^–6^). We further performed stratification analyses by drinking status, smoking status, and age group. We found that the association of all five of the miRNAs with MN frequency remained significant in drinkers (all *p* < 0.005), and most associations were significantly stronger in drinkers compared with nondrinkers (all *p*_interaction_ < 0.015), except for the association between miR-142-5p and MN frequency (*p*_interaction_ = 0.288) (Table 4). Although the associations between miRNA expression and MN frequency also remained significant in smokers and workers between 41 and 60 years of age (all *p* < 0.025), most associations were not significantly different between the corresponding subgroups (all *p*_interaction_ > 0.05), except for the association of miR-27a-3p with MN frequency between smoking strata (*p*_interaction_ = 0.049) (see Supplemental Material, Table S5).

**Table 4:** Association between miRNA expression and MN frequency (as the dependent variable) [FR (95% CI)].

miRNA^*a*^	All samples (*n* = 365)	*p*^*b*^	Nondrinkers (*n* = 205)	*p*^*b*^	Drinkers (*n* = 160)	*p*^*b*^	*p*_interaction_^*c*^
miR-24-3p	1.152 (1.086, 1.222)	2.59 × 10^–6^	1.035 (0.954, 1.122)	0.407	1.293 (1.182, 1.415)	2.34 × 10^–8^	3.36 × 10^–4^
miR-27a-3p	1.093 (1.032, 1.157)	0.002	1.021 (0.944, 1.103)	0.608	1.180 (1.080, 1.290)	2.47 × 10^–4^	0.007
miR-142-5p	1.102 (1.041, 1.166)	8.59 × 10^–4^	1.076 (0.996, 1.163)	0.064	1.120 (1.027, 1.221)	0.010	0.288
miR-28-5p	1.147 (1.083, 1.216)	3.69 × 10^–6^	1.050 (0.965, 1.144)	0.259	1.236 (1.137, 1.344)	6.68 × 10^–7^	0.013
miR-150-5p	1.092 (1.033, 1.155)	0.002	0.997 (0.917, 1.082)	0.936	1.172 (1.084, 1.266)	6.16 × 10^–5^	0.009
^***a***^Values were log_2_-transformed. ^***b***^Poisson regression analysis with adjustment for age, smoking status, pack-years of smoking, working years, workplace, BMI, ΣOH-PAHs, BPDE–Alb adducts, and/or drinking status when appropriate. ^***c***^*p*_interaction_ was calculated by entering the interaction term (miRNA*drinking status) into Poisson regression models, with adjustment for age, smoking status, pack-years of smoking, working years, workplace, BMI, ΣOH-PAHs, and BPDE–Alb adducts.							

*Enriched biological functions of the target genes*. To explore the potential functions of these five miRNAs, we predicted their target genes. There were 733 putative targets for miR-24-3p, 452 for miR-27a-3p, 66 for miR-142-5p, 432 for miR-28-5p, and 721 for miR-150-5p. GO function enrichment analysis showed that these target genes were mainly involved in 13 enriched biological functions (*p* < 0.002), including the cellular physiological process, external stimulus response, enzyme activity regulation, and metabolism (Table 5).

**Table 5:** Enriched biological functions of the target genes of five identified miRNAs (miR-24-3p, miR-27a-3p, miR-142-5p, miR-28-5p, and miR-150-5p).

ID	Description	*p*-Value
GO:0030036	Actin cytoskeleton organization	1.49 × 10^–4^
GO:0030029	Actin filament-based process	2.78 × 10^–4^
GO:0006468	Protein amino acid phosphorylation	3.44 × 10^–4^
GO:0065009	Regulation of molecular function	5.46 × 10^–4^
GO:0009605	Response to external stimulus	5.78 × 10^–4^
GO:0007242	Intracellular signaling cascade	6.37 × 10^–4^
GO:0065008	Regulation of biological quality	7.81 × 10^–4^
GO:0051347	Positive regulation of transferase activity	1.00 × 10^–3^
GO:0010646	Regulation of cell communication	1.13 × 10^–3^
GO:0007243	Protein kinase cascade	1.72 × 10^–3^
GO:0050790	Regulation of catalytic activity	1.73 × 10^–3^
GO:0051017	Actin filament bundle formation	1.88 × 10^–3^
GO:0044267	Cellular protein metabolic process	1.95 × 10^–3^

## Discussion

To the best of our knowledge, this is the first study to explore associations between PAH exposure and miRNA expression, and between the same miRNAs and MN frequency, in an occupational population. Our genome-wide miRNA sequencing revealed that miRNA expression profiles were different between two occupational groups with high and low PAH exposure levels, with most miRNAs significantly down-regulated in the high-exposure group compared with the low-exposure controls. In a detailed validation study, we identified five miRNAs that were associated with at least one of the PAH exposures, including four that were negatively associated with urinary noncarcinogenic 4-hydroxyphenanthrene and/or plasma carcinogenic BPDE–Alb adducts (miR-24-3p, miR-27a-3p, miR-142-5p, and miR-28-5p) and one that was positively associated with exposure to three different urinary noncarcinogenic OH-PAHs and with urinary ΣOH-PAHs (miR-150-5p). The same miRNAs also were associated with chromosome damage, as reflected by higher MN frequency, with stronger associations among drinkers than nondrinkers.

Many studies have reported evidence suggesting that environmental stimuli can induce changes in miRNA expression (Bollati et al. 2010; Jardim et al. 2009; Schembri et al. 2009). The present study provides novel information about associations of PAH exposures with plasma miRNA expression in exposed workers. Our genome-wide miRNA sequencing and subsequent validation revealed that most miRNAs were negatively associated with PAH exposure levels, which is similar to the results reported in prior studies regarding the effects of PAH-rich cigarette smoke on miRNA expression patterns (De Flora et al. 2012). Izzotti et al. (2009) reported that cigarette smoke mainly resulted in a remarkable down-regulation of miRNA expression in rat lung. Schembri et al. (2009) observed 28 miRNAs that were differentially expressed in the bronchial airway epithelium in smokers, with most being down-regulated. Moreover, miRNA expression is generally down-regulated in different types of cancer, including lung cancer (Lu et al. 2005).

In the present study, miR-24-3p, miR-27a-3p, miR-142-5p, and miR-28-5p were all negatively associated with urinary 4-hydroxyphenanthrene concentrations and/or plasma BPDE–Alb adducts, and positively associated with MN frequency. These miRNAs have been reported to regulate genes that could protect against adverse effects of PAH exposures. miR-24-3p has been reported to negatively regulate *H2AX*, which is crucial in double-stranded break repair; thus, reduced expression of this gene might increase cellular sensitivity to DNA-damaging agents and genomic instability (Lal et al. 2009; Wang and Taniguchi 2013). In addition, miR-24-3p has also been reported to negatively regulate *ARNT* (aryl hydrocarbon receptor nuclear translocator), the protein product of which forms a heterodimer with the aryl hydrocarbon receptor that mediates PAH responses, and to down-regulate the metabolism gene *CYP1A1* (cytochrome P450 1A1) (Oda et al. 2012). miR-27a-3p has been proposed to operate with miR-24-3p in a cooperative cluster; it can be down-regulated by reactive oxygen species (Pathi et al. 2011), and it is a key regulator of *TFIIH*, which displays activities involved in DNA repair processes (Portal 2011). miR-27a-3p down-regulation may elevate TFIIH and DNA repair capacity and thus decrease chromosome damage. miR-142-5p is repressed in lung cancer (Liu et al. 2009) and down-regulated after exposure to benzo[*a*]pyrene (Halappanavar et al. 2011). miR-142-5p down-regulation in healthy CD4^+^ T cells can lead to up-regulation of *SAP* (SLAM-associated protein) expression and increase T-cell function and IgG production (Ding et al. 2012); thus, it may protect individuals against the deleterious effects of PAHs. miR-28-5p is linked to frequent chromosomal alterations (Wilting et al. 2013); it negatively regulates *Nrf2* (NF-E2-related factor 2), the protein product of which is an important transcription factor that regulates the expression of detoxifying enzymes (Yang et al. 2011). miR-28-5p down-regulation may elevate the expression of *Nrf2* and detoxifying enzymes, and may protect cells from carcinogen-induced DNA damage.

In addition, we found that miR-150-5p expression, which was increased in association with several biomarkers of internal noncarcinogenic PAH exposure, was associated with higher MN frequency. However, Halappanavar et al. (2011) found that miR-150-5p was down-regulated in the lung of rats exposed to benzo[*a*]pyrene. miR-150-5p is a key regulator of *c-Myb*, which is important for immune cell differentiation and activation, and miR-150 deficiency can lead to enhanced immune response (Xiao et al. 2007). Thus, miR-150-5p up-regulation may decrease immune response to PAH exposure and make individuals more vulnerable to the deleterious effects of PAHs.

The stratification analyses in our study showed that the associations between miRNAs and MN frequency were more prominent in drinkers. These results provided some clues that can be used for more detailed risk assessment.

Our study has several strengths. First, to identify PAH-associated miRNAs, we screened and compared hundreds of miRNAs in pooled plasma samples from high- and low-exposure groups, and then we validated several miRNAs to reduce the false-positive probability. Second, we systematically evaluated the associations of miRNAs with noncarcinogenic and carcinogenic PAH internal exposure biomarkers. For our study, we recruited workers who had been regularly exposed to PAH-rich emissions for at least 1 year, with their major PAH exposure sources and concentrations showing little fluctuation. Thus, it was reasonable to use OH-PAHs and BPDE–Alb adducts as biomarkers of chronic PAH exposure in this population (Sobus et al. 2009). Moreover, BPDE–Alb adducts cannot be repaired and have a mean residence time of 28 days, which is sufficiently long to dampen the day-to-day variability in exposure levels (Chung et al. 2010). Besides, our study subjects had been working in the same factory, minimizing the confounding effects of other PAH exposure sources from the day-to-day environment, socieconomic factors, and other characteristics.

However, because our study is a cross-sectional study in which we measured biomarkers of PAH exposures, miRNA expression levels, and MN frequency at the same time point, it is not possible to determine whether differences in miRNA expression preceded or followed PAH exposures or chromosome damage. Moreover, we validated only a subset of the miRNAs found in the discovery stage, and other miRNAs should be further validated.

## Conclusions

We identified five potentially PAH-associated miRNAs in plasma, and the same miRNAs were associated with a marker of chromosome damage in coke oven workers, suggesting that miRNAs might be a novel mechanism mediating the effects of PAH exposure on chromosome damage. Further studies are warranted to verify our findings and determine the underlying mechanisms.

## Supplemental Material

(342 KB) PDF
