# Supplementary material for: Plasma microRNA Expression and Micronuclei Frequency in Workers Exposed to Polycyclic Aromatic Hydrocarbons
Source: Environ Health Perspect. 2014 Mar 14;122(7):719–25. doi: 10.1289/ehp.1307080 (PMC4080537; doi:10.1289/ehp.1307080)
Supplement: (342 KB) PDF [file ehp.1307080.s001.pdf]

Supplemental Material

Plasma microRNA Expression and Micronuclei Frequency in Workers Exposed to Polycyclic Aromatic Hydrocarbons

Qifei Deng, Suli Huang, Xiao Zhang, Wangzhen Zhang, Jing Feng, Tian Wang, Die Hu, Lei Guan, Jun Li, Xiayun Dai, Huaxin Deng, Xiaomin Zhang, and Tangchun Wu

Table of Contents **Page**

Table S1. Partial correlation coefficients between 10 urinary OH-PAHs, ΣOH-PAHs, and plasma BPDE-Alb adducts in 391 healthy male coke oven workers **2**

Table S2. LOQ and percentage of quantifiable samples of each biomarker **3**

Table S3. Associations between PAH exposure levels and MN frequency (as the dependent variable) in the validation stage **4**

Table S4. Associations of the expression levels of five PAH-associated miRNAs (as the dependent variable) with smoking status, age, and drinking status in the validation stage [standardized β (95% CI)]. **5**

Table S5. Associations between miRNA expression and MN frequency (as the dependent variable) in workers with different smoking status or age groups [FR (95% CI)]. **6**

Table S1. Partial correlation coefficients[a] between 10 urinary OH-PAHs, ΣOH-PAHs, and plasma BPDE-Alb adducts in 391 healthy male coke oven workers.

**PAH internal exposure biomarkers**[b]	**1-hydroxy-naphthalene**	**2-hydroxy-naphthalene**	**2-hydroxy-fluorene**	**9-hydroxy-fluorene**	**1-hydroxy-phenanthrene**	**2-hydroxy-phenanthrene**	**3-hydroxy-phenanthrene**	**4-hydroxy-phenanthrene**	**9-hydroxy-phenanthrene**	**1-hydroxy-pyrene**	**ΣOH-PAHs**	**BPDE-Alb**	**Σ Partial *r***[c]
1-hydroxynaphthalene		0.834**	0.473**	0.332**	0.541**	0.596**	0.480**	0.189**	0.614**	0.686**	0.862**	0.156*	5.763
2-hydroxynaphthalene	0.834**		0.442**	0.255**	0.497**	0.477**	0.408**	0.140*	0.519**	0.597**	0.793**	0.107*	5.069
2-hydroxyfluorene	0.473**	0.442**		0.364**	0.263**	0.621**	0.575**	0.132*	0.498**	0.407**	0.579**	-0.004	4.350
9-hydroxyfluorene	0.332**	0.255**	0.364**		0.253**	0.267**	0.078	0.330**	0.366**	0.282**	0.485**	0.065	3.077
1-hydroxyphenanthrene	0.541**	0.497**	0.263**	0.253**		0.436**	0.213**	0.130*	0.564**	0.653**	0.695**	0.074	4.319
2-hydroxyphenanthrene	0.596**	0.477**	0.621**	0.267**	0.436**		0.718**	0.053	0.690**	0.644**	0.672**	0.057	5.231
3-hydroxyphenanthrene	0.480**	0.408**	0.575**	0.078	0.213**	0.718**		0.017	0.464**	0.404**	0.483**	0.068	3.908
4-hydroxyphenanthrene	0.189**	0.140*	0.132*	0.330**	0.130*	0.053	0.017		0.126*	0.179**	0.305**	0.092	1.693
9-hydroxyphenanthrene	0.614**	0.519**	0.498**	0.366**	0.564**	0.690**	0.464**	0.126*		0.673**	0.736**	0.108*	5.358
1-hydroxypyrene	0.686**	0.597**	0.407**	0.282**	0.653**	0.644**	0.404**	0.179**	0.673**		0.852**	0.246**	5.623
ΣOH-PAHs	0.862**	0.793**	0.579**	0.485**	0.695**	0.672**	0.483**	0.305**	0.736**	0.852**		0.201**	6.663
BPDE-Alb	0.156*	0.107*	-0.004	0.065	0.074	0.057	0.068	0.092	0.108*	0.246**	0.201**		1.170

[a]Partial correlation analysis with adjustment for age, smoking status, pack-years of smoking, drinking status, working years, workplace, and BMI. [b]Ln-transformed. [c]The sum of the partial correlation coefficients for each PAH internal exposure biomarker. **P* < 0.05; ***P* < 0.001.

Table S2. LOQ and percentage of quantifiable samples of each biomarker.

PAH internal exposure biomarkers[a]	LOQ	n	Q%
1-hydroxynaphthalene	0.9	364	99.73
2-hydroxynaphthalene	0.9	364	99.73
2-hydroxyfluorene	0.1	359	98.36
9-hydroxyfluorene	0.2	360	98.63
1-hydroxyphenanthrene	0.3	363	99.45
2-hydroxyphenanthrene	0.1	363	99.45
3-hydroxyphenanthrene	0.2	360	98.63
4-hydroxyphenanthrene	0.1	356	97.53
9-hydroxyphenanthrene	0.2	362	99.18
1-hydroxypyrene	0.5	365	100
6-hydroxychrysene	1.4	0	0
3-hydroxybenzo[a]pyrene	1.0	0	0
BPDE-Alb	1.0	343	93.97

[a]The unit for OH-PAHs is μmol/mmol creatine, and the unit for BPDE-Alb adducts is ng/mg albumin.

Table S3. Associations between PAH exposure levels and MN frequency (as the dependent variable) in the validation stage.

**PAH internal exposure biomarkers[a]**	**FR (95% CI)**	***P*[b]**
1-hydroxynaphthalene	1.035 (0.976, 1.099)	0.253
2-hydroxynaphthalene	1.022 (0.959, 1.090)	0.500
2-hydroxyfluorene	1.060 (1.001, 1.125)	0.049
9-hydroxyfluorene	1.064 (1.005, 1.128)	0.034
1-hydroxyphenanthrene	1.036 (0.977, 1.100)	0.237
2-hydroxyphenanthrene	1.033 (0.977, 1.094)	0.258
3-hydroxyphenanthrene	1.020 (0.965, 1.078)	0.491
4-hydroxyphenanthrene	1.085 (1.025, 1.150)	0.006
9-hydroxyphenanthrene	1.020 (0.965, 1.078)	0.491
1-hydroxypyrene	1.051 (0.992, 1.113)	0.091
ΣOH-PAHs	1.072 (1.012, 1.135)	0.017
BPDE-Alb	1.134 (1.078, 1.192)	8.41×10^{-7}

Abbreviations: FR: frequency ratio.

[a]Ln-transformed. [b]Poisson regression analysis with adjustment for age, smoking status, pack-years of smoking, drinking status, working years, workplace, and BMI.

Table S4. Associations of the expression levels of five PAH-associated miRNAs (as the dependent variable) with smoking status, age, and drinking status in the validation stage [standardized β (95% CI)].

**miRNA**[a]	**Drinking status**	***P***[b]	**Smoking status**	***P***[c]	**Age**	***P***[d]
miR-24-3p	-0.273 (-1.043, 0.497)	0.486	0.429 (-0.407, 1.266)	0.313	-0.223 (-1.175, 0.709)	0.627
miR-27a-3p	-0.236 (-0.803, 0.330)	0.412	0.500 (-0.120, 1.119)	0.114	-0.162 (-0.855, 0.531)	0.646
miR-142-5p	-0.393 (-1.009, 0.224)	0.211	0.579 (-0.094, 1.252)	0.091	0.129 (-0.627, 0.886)	0.737
miR-28-5p	0.000 (-0.634, 0.633)	1.000	0.162 (-0.532, 0.855)	0.647	-0.049 (-0.824, 0.726)	0.900
miR-150-5p	0.224 (-0.147, 0.595)	0.236	0.037 (-0.368, 0.441)	0.859	-0.051 (-0.504, 0.401)	0.824

[a]Log2-transformed. [b]Multivariable linear regression analysis with adjustment for age, smoking status, pack-years of smoking, working years, workplace, BMI, ΣOH-PAHs, and BPDE-Alb adducts. [c]Multivariable linear regression analysis with adjustment for age, drinking status, working years, workplace, BMI, ΣOH-PAHs, and BPDE-Alb adducts. [d]Multivariable linear regression analysis with adjustment for smoking status, pack-years of smoking, drinking status, working years, workplace, BMI, ΣOH-PAHs, and BPDE-Alb adducts.

Table S5. Associations between miRNA expression and MN frequency (as the dependent variable) in workers with different smoking status or age groups [FR (95% CI)].

**miRNAs**[a]	**Nonsmokers (n=113)**	***P***[b]	**Smokers (n=252)**	***P***[b]	$P_{interaction}$[c]	**20-40 years old (n=164)**	***P***[d]	**41-60 years old (n=201)**	***P***[d]	$P_{interaction}$[e]
miR-24-3p	1.066 (0.966, 1.177)	0.204	1.190 (1.106, 1.282)	$3.51\times10^{-6*}$	0.098	1.111 (1.013, 1.219)	0.025	1.174 (1.085, 1.271)	$7.56\times10^{-5*}$	0.684
miR-27a-3p	1.011 (0.919, 1.111)	0.826	1.142 (1.062, 1.229)	$3.80\times10^{-4*}$	0.049	1.070 (0.977, 1.172)	0.145	1.097 (1.017, 1.185)	0.018	0.875
miR-142-5p	1.118 (1.009, 1.240)	0.034	1.094 (1.021, 1.173)	0.011	0.814	1.088 (0.993, 1.191)	0.071	1.092 (1.013, 1.178)	0.023	0.870
miR-28-5p	1.070 (0.962, 1.192)	0.214	1.173 (1.094, 1.258)	$7.38\times10^{-6*}$	0.187	1.111 (1.015, 1.217)	0.022	1.167 (1.078, 1.264)	$1.49\times10^{-4*}$	0.737
miR-150-5p	1.068 (0.960, 1.188)	0.224	1.099 (1.028, 1.174)	0.005^{*}	0.684	1.063 (0.977, 1.155)	0.155	1.101 (1.019, 1.188)	0.014	0.860

Abbreviations: FR: frequency ratio.

[a]Log2-transformed. [b]Poisson regression analysis with adjustment for age, drinking status, working years, workplace, BMI, ΣOH-PAHs, and BPDE-Alb adducts. [c]$P_{interaction}$ was calculated by entering the interaction term (miRNA*smoking status) into Poisson regression models, with adjustment for age, drinking status, working years, workplace, BMI, ΣOH-PAHs, and BPDE-Alb adducts. [d]Poisson regression analysis with adjustment for smoking status, pack-years of smoking, drinking status, working years, workplace, BMI, ΣOH-PAHs, and BPDE-Alb adducts. [e]$P_{interaction}$ was calculated by entering the interaction term (miRNA*age group) into Poisson regression models, with adjustment for smoking status, pack-years of smoking, drinking status, working years, workplace, BMI, ΣOH-PAHs, and BPDE-Alb adducts.
